# Microbial Contamination of Date *Rutab* Collected from the Markets of Al-Hofuf City in Saudi Arabia

**DOI:** 10.1100/2012/124892

**Published:** 2012-04-24

**Authors:** Siddig H. Hamad, Farag A. Saleh, Mutlag M. Al-Otaibi

**Affiliations:** Department of Food Science and Nutrition, College of Agricultural and Food Sciences, King Faisal University, P.O. Box 420, Al-Hofuf 31982, Saudi Arabia

## Abstract

The microbial contamination of 60 samples from six date cultivars in the *rutab* stage purchased from different retail outlets in AL-Hofuf City, Saudi Arabia was studied. All samples were found contaminated with aerobic mesophilic bacteria at loads in the order 10^2^ to 10^5^ cfu/cm^2^ with some significant differences among varieties that can be attributed to differences in the weather conditions during *rutab *season. Also all samples, except only one, were contaminated with molds and yeasts at loads in the order 10^2^ to 10^3^ cfu/cm^2^. Potentially pathogenic *Staphylococcus aureus* was detected in 57 samples and *A. flavus/parasiticus* in 13 samples, while coliforms were detected in 39 samples.

## 1. Introduction

Date (*Phoenix dactylifera* L.) is mainly produced in Middle Eastern and North African countries. It is considered an important subsistence food crop in most of these countries, in addition to its high cultural and religious significance. Date fruit production has constantly increased worldwide over the last four decades from 1.8 million tons in 1963 to 6.7 millions in 2003 [1]. Saudi Arabia, with about 830 thousand tons annual production, ranks as the third largest date producer in the world [1]. *Rutab* (an Arabic name) is a stage of maturity in which the date fruit can be consumed as human food. It is a fresh product that contains 35 to 40% water and 45 to 48% sugars (dry matter basis) [2]. Because of its high moisture content,* rutab* is highly susceptible to microbial growth and spoilage, especially when poor hygienic practices during handling lead to heavy microbial contamination. Microbiological studies on dates are limited, especially in the main countries of production [3–9]. No published work on the microbial contamination of *rutab* was found.

This present study was undertaken to investigate the microbial contamination of *rutab *from different date cultivars grown in the Gulf Region in Saudi Arabia.

## 2. Materials and Methods

### 2.1. Rutab Samples

The *rutab* samples were purchased from 10 different retail outlets in Al-Hofuf City and represented the most popular six date cultivars grown in the region: *Khulas, Um-Ruhaim, Hilali*, *Shahal, Tiar, *and* Megnaz*. A total of 60 samples were collected at weekly interval, 10 samples from each cultivar at each sampling time, and microbial analysis was carried out on the same day.

### 2.2. Microbiological Analysis

Four fruits from each *rutab *sample were transferred into sterile stomacher bags, 50 mL sterile peptone water (CM0009, Oxoid, Basingstoke, UK) added, and the fruits washed manually for 2 minutes in this water, then aliquots (1.0 or 0.1 mL) were plated in duplicate as 10-fold dilutions in peptone water (surface area of the 4 fruits was then measured). Three replicates were analyzed for each sample and the average microbial loads calculated and reported as colony-forming units (cfu) per cm^2^. Aerobic mesophilic bacteria were enumerated on plate count agar (CM0325, Oxoid) incubated at 30°C for 2 to 3 days. Coliforms were determined on violet red bile agar plates (VRBA, CM0107, Oxoid) incubated at 37°C for 24 hours. Round, purple-red colonies (0.5–2 mm diameter) surrounded by purple-red haloes on VRBA plates were counted as coliforms. The presence of *Escherichia coli* O157 among coliforms detected on VRBA plates was tested using the *Escherichia coli* O157 Latex Test (DR0620, Oxoid). Yeasts and molds were counted on potato dextrose agar plates (CM0139, Oxoid) incubated at 30°C for 3 days. *Staphylococcus aureus* was enumerated on *Staphylococcus* medium no. 110 (CM0145, Oxoid) incubated at 35–37°C for 24–48 hours (colonies with yellow orange pigment and clearing zone around them) and identified using the Staphylase Test (DR0595, Oxoid). *Aspergillus flavus/parasiticus* was detected and enumerated on *Aspergillus flavus*-*parasiticus* agar (CM0731, Oxoid) incubated at 30°C for 48 hours. Colonies that form bright orange-yellow pigments on the colony reverse were counted as *Aspergillus flavus/parasiticus.*

### 2.3. Statistical Analysis

A multiple comparison statistical procedure using Fisher's least significant difference test (SAS software, version 6.11) was used to determine the significance of differences among *rutab* cultivars to microbial spoilage at significance levels of *P* ≤ 0.05 [10].

## 3. Results and Discussion

All *rutab* samples from the six date varieties tested were found contaminated with aerobic mesophilic bacteria at loads in the order 10^2^ to 10^5^ cfu/cm^2^ (Table 1). The levels of contamination with these microorganisms are indices of the general microbiological quality and give idea about the expected shelf lives of the foods concerned. The highest levels of contamination with these bacteria were found in *Hilali*. Five samples from this variety had loads of 1.6 × 10^5^ to 2.3 × 10^5^, one sample a load of 3.8 × 10^4^, and 4 samples 2.9 × 10^3^ to 6.9 × 10^3^ cfu/cm^2^. *Shahal* samples had generally lower loads of contamination than *Hilali*, with one sample having 2.6 × 10^5^, 6 samples 1.5 × 10^4^ to 4.5 × 10^4^, and 3 samples 1.5 × 10^3^ to 9.1 × 10^3^ cfu/cm^2^. The loads of the other 4 varieties were still lower and comparable to each other. *Um Ruhaim* had loads of 1.8 × 10^4^ to 4.4 × 10^4^ in 4 samples, and 1.3 × 10^3^ to 5.6 × 10^3^ in 6 samples, *Khulas* had 1.3 × 10^4^ to 4.2 × 10^4^ in 4 samples, and 1.5 × 10^3^ to 4.3 × 10^3^ cfu/cm^2^in 6 samples, *Tiar* had 1.0 × 10^5^ in one sample, 2.6 × 10^4^ in one sample, and 1.1 × 10^3^ to 6.5 × 10^3^ cfu/cm^2^ in 8 samples, while *Megnaz *had 1.1 × 10^4^ to 1.9 × 10^4^ in 3 samples and 1.1 × 10^3^ to 8.0 × 10^3^ cfu/cm^2^ in 7 samples. The level of *Hilali* contamination was significantly higher than that of *Khulas, Um Ruhaim*, *Tiar, *and *Megnaz;* it was also higher than the level of *Shahal*, but the difference was not significant. On the other hand, the level of contamination of *Shahal* was higher than that of *Khulas*,* Um Ruhaim, Tiar,* and *Megnaz*, but the difference was not significant. The levels of contamination of *Khulas*, *Um-Ruhaim*, *Tiar* and *Megnaz* were quite similar (Table 1). The differences in the levels of contamination with aerobic mesophilic bacteria among these *rutab* samples may be attributed to differences in environmental temperatures during *rutab* season. *Khulas*, *Um Ruhaim, Tiar,* and *Megnaz* are early maturing varieties and they were sampled and analyzed by the end of August where the temperatures in Saudi Arabia were above 40°C, thus the spread of mesophilic bacteria is expected to be limited. *Shahal* matures in mid-August and was sampled and analyzed mid-September, where the temperatures start to drop below 40°C, while *Hilali* is a late maturing variety and its samples in this study were collected and analyzed in October where the temperatures were in the thirties, and hence the mesophilic bacteria are supposed to dominate in the environment.

Almost all samples were contaminated with varying loads of molds and yeasts (Table 2), which are generally spoilage organisms, although some of them could be pathogenic. *Shahal* and *Hilali* showed the highest levels of contaminations with molds and yeasts. One *Shahal* sample had 1.9 × 10^4^, 2 samples 2.3 × 10^3^ and 4.6 × 10^3^, and 7 samples 1.7 × 10^2^ to 7.8 × 10^2^ cfu/cm^2^. One *Hilali* sample had 1.1 × 10^4^, 3 samples 1.2 × 10^3^ to 5.9 × 10^3^, and 6 samples 1.1 × 10^2^ to 4.7 × 10^2^ cfu/cm^2^. Two *Um Ruhaim* samples had 1.8 × 10^3^ and 2.0 × 10^3^, and 8 samples 2.0 × 10^2^ to 6.9 × 10^2^, cfu/cm^2^, while 3 *Khulas* samples had 1.0 × 10^3^ to 1.2 × 10^3^, 6 samples 2.1 × 10^2^ to 4.8 × 10^2^ cfu/cm^2^, and one sample undetectable level. In case of *Tiar*, only one sample showed a level of contamination of 1.5 × 10^3^ and the contamination of the other 9 samples was in the range 1.3 × 10^2^ to 3.3 × 10^2^ cfu/cm^2^. The contamination of *Megnaz *samples was 1.4 × 10^3^ in one sample and 1.3 × 10^2^ to 5.2 × 10^2^ cfu/cm^2^ in 9 samples. Contamination of *Khulas*, *Um Ruhaim, Tiar,* and *Megnaz *with molds and yeasts was generally lower than that of *Hilali *and *Shahal*, but the differences were not significant (Table 2).

Microbial contaminations in the order 10^5^ cfu/g·cm^2^·mLa can be regarded as high since signs of microbial spoilage start to appear in most foods at loads of about 10^6^ cfu/g·cm^2^·mL [11]. *Rutab* with such high microbial contamination levels is expected to have a short shelf life if kept at room temperature. This high level of contamination was mainly found in *Hilali* where 5 of the 10 samples tested contained more than 10^5^ cfu/cm^2^ (Table 1). 

With respect to pathogenic microorganisms, all samples of *Khulas, Um-Ruhaim, Shahal, Hilali, *and *Megnaz *and 7 samples of *Tiar *were found contaminated with the potential food poisoning bacterium *Staphylococcus aureus* (Table 3). The highest level of contamination with this bacterium was in *Shahal*, followed by *Um Ruhaim*, then *Khulas* but the differences between the three were not significant. The level of contamination of *Hilali* and *Megnaz* was significantly lower than that of *Shahal*, *Um Ruhaim*, and *Khulas* and the level of contamination of *Tiar* was still lower than all other samples with significant difference from all of them (Table 3). *Rutab* in Saudi Arabia is harvested manually; thus it seems that contamination of the date fruits with this bacterium came mainly from workers, and differences in the degree of contamination may be attributed to differences in the hygienic status of these workers. *Staphylococcus aureus* can infect and grow in wounds on the hands of workers inevitably caused during harvesting by the sharp and strong ends of the leaves of the palm tree. 

Coliforms were found in 39 out of the 60 samples analyzed, that is, 65% of the samples studied (Table 4). Their rate of occurrence was similar in 5 cultivars ranging between 6 and 8 contaminated samples, while in *Khulas* only 4 samples were found contaminated. The loads were relatively low reaching the range of 10^2^ cfu/g in only 4 samples. The source of contamination was most likely the workers who harvested *rutab* manually. No *Escherichia coli* O157 was detected among these coliforms.

The potential mycotoxin producing molds* A. flavus *and* A. parasiticus* were found in 13 out of the 60 samples analyzed, that is, 21.7% contaminated samples (results not shown). They were detected in 2 *Khulas*, 3 *Um-Ruhaim*, 4 *Shahal,* and 4 *Hilali *but none in *Tiar* or *Megnaz* samples. The loads were generally low; the highest was 60 cfu/cm^2^ in one sample. In spite of their low loads in the samples, if these molds find appropriate conditions for growth and mycotoxin production, the situation can be hazardous. 

Microbiological information on the quality of dates is limited, especially in the main countries of production. The level of contamination of most *rutab* samples in this present study may be considered satisfactory in comparison to levels reported for other fruits such as grapes with 10^7^ yeasts g^−1^, and strawberries, raspberries, and blackberries with 10^4^ to 10^6^ g^−1^ yeasts, up to 10^4^ g^−1^ molds, and 10^5^ to 10^6^ g^−1^ bacteria [12]. The few reports on the microbiology of date fruits were mainly on *tamr* (Arabic name for fully mature date fruits). A study on the microbial spoilage of *rutab* showed that spoilage is mainly caused by lactic acid bacteria, yeasts, and molds [7], while the microbial spoilage of *tamr* is caused by yeasts, molds, and bacteria [8].* Tamr* with moisture level raised to 27–30%, inoculated with yeast suspensions, and incubated at 25°C was spoiled in 5 days [5]. Colony counts of soft dates in the *tamr* stage of the order 10^4^ cfu/g lactic acid bacteria and 10^2^ cfu/g yeasts were reported [9]. Loose dates were found contaminated with *S. aureus* and aerobic colony counts of 6.3 × 10^5^ cfu/g [6]. Date (*tamr*) samples purchased in stores within Greater Glasgow were found contaminated with up to 10^4^ cfu/g aerobic mesophilic bacteria, up to 3.4 × 10^3^ cfu/g coliforms, and up to 6.9 × 10^4^ cfu/g yeasts and molds [4]. These authors also detected *A. flavus/parasiticus*, *Bacillus cereus*, *E. coli,* and *S. aureus* in some samples. No published work on the microbial contamination of *rutab* was found in the literature cited.

## Figures and Tables

**Table 1:** Counts of aerobic mesophilic bacteria contaminating *rutab* samples (cfu/cm^2^).

Sample	*Khulas*	*Um-Ruhaim*	*Shahal*	*Hilali*	*Tiar*	*Megnaz*
1	1.3 × 10^4^	1.8 × 10^4^	4.5 × 10^4^	4.1 × 10^3^	4.3 × 10^3^	5.2 × 10^3^
2	2.2 × 10^3^	4.4 × 10^4^	3.8 × 10^4^	3.2 × 10^3^	3.0 × 10^3^	1.9 × 10^4^
3	3.2 × 10^3^	2.8 × 10^3^	1.5 × 10^4^	6.9 × 10^3^	6.5 × 10^3^	2.0 × 10^3^
4	1.5 × 10^3^	5.9 × 10^3^	1.8 × 10^4^	3.8 × 10^4^	1.1 × 10^3^	1.1 × 10^4^
5	4.2 × 10^4^	2.4 × 10^4^	1.5 × 10^3^	2.3 × 10^5^	3.4 × 10^3^	1.1 × 10^3^
6	7.3 × 10^2^	2.0 × 10^3^	2.6 × 10^5^	2.9 × 10^3^	2.6 × 10^4^	6.7 × 10^3^
7	4.3 × 10^3^	3.9 × 10^3^	3.7 × 10^3^	1.9 × 10^5^	5.2 × 10^3^	1.8 × 10^4^
8	2.0 × 10^4^	3.3 × 10^4^	9.1 × 10^3^	2.0 × 10^5^	5.0 × 10^3^	8.0 × 10^3^
9	4.2 × 10^4^	1.3 × 10^3^	2.6 × 10^4^	1.6 × 10^5^	1.5 × 10^3^	4.8 × 10^3^
10	3.7 × 10^3^	5.5 × 10^3^	1.6 × 10^4^	1.8 × 10^5^	1.0 × 10^5^	5.8 × 10^3^

Mean*	3.77^b^	3.88^b^	4.24^ab^	4.54^a^	3.75^b^	3.78^b^

*log_10_; means with the same letter are not significantly different (*P* ≤ 0.05).

**Table 2:** Counts of yeasts and molds contaminating *rutab* samples (cfu/cm^2^).

Sample	*Khulas*	*Um-Ruhaim*	*Shahal*	*Hilali*	*Tiar*	*Megnaz*
1	4.8 × 10^2^	2.0 × 10^3^	7.8 × 10^2^	2.5 × 10^2^	2.6 × 10^2^	5.2 × 10^2^
2	3.3 × 10^2^	2.4 × 10^2^	1.9 × 10^4^	1.1 × 10^4^	2.3 × 10^2^	2.2 × 10^2^
3	2.1 × 10^2^	3.0 × 10^2^	1.7 × 10^2^	4.7 × 10^2^	2.2 × 10^2^	1.7 × 10^2^
4	1.2 × 10^3^	2.0 × 10^2^	3.7 × 10^2^	5.9 × 10^3^	1.5 × 10^3^	2.6 × 10^2^
5	n.d.	6.9 × 10^2^	2.2 × 10^2^	1.2 × 10^3^	1.3 × 10^2^	1.4 × 10^2^
6	1.1 × 10^3^	3.2 × 10^2^	4.6 × 10^3^	3.4 × 10^2^	3.3 × 10^2^	1.4 × 10^3^
7	2.1 × 10^2^	1.8 × 10^3^	2.3 × 10^3^	1.2 × 10^3^	2.2 × 10^2^	2.2 × 10^2^
8	4.5 × 10^2^	3.4 × 10^2^	5.2 × 10^2^	1.8 × 10^2^	2.6 × 10^2^	1.3 × 10^2^
9	2.1 × 10^2^	5.3 × 10^2^	3.3 × 10^2^	2.3 × 10^2^	1.5 × 10^2^	3.2 × 10^2^
10	1.0 × 10^3^	4.5 × 10^2^	3.9 × 10^2^	1.1 × 10^2^	1.7 × 10^2^	2.4 × 10^2^

Mean*	2.39^a^	2.70^a^	2.92^a^	2.82^a^	2.41^a^	2.43^a^

*log_10_; means with the same letter are not significantly different (*P* ≤ 0.05). n.d.: not detected.

**Table 3:** Counts of *Staphylococcus aureus* contaminating *rutab* samples (cfu/cm^2^).

Sample	*Khulas*	*Um-Ruhaim*	*Shahal*	*Hilali*	*Tiar*	*Megnaz*
1	1.9 × 10^2^	1.5 × 10^3^	3.5 × 10^2^	41	8	39
2	1.2 × 10^2^	2.4 × 10^2^	3.0 × 10^2^	52	nd	86
3	2.4 × 10^2^	27	1.6 × 10^3^	99	15	24
4	77	64	3.8 × 10^2^	1.4 × 10^2^	5	58
5	58	2.6 × 10^2^	1.0 × 10^3^	4.5 × 10^2^	17	30
6	1.5 × 10^2^	1.0 × 10^3^	40	66	86	1.3 × 10^2^
7	1.1 × 10^3^	28	3.3 × 10^2^	1.3 × 10^2^	13	2.2 × 10^2^
8	3.5 × 10^2^	2.7 × 10^2^	2.9 × 10^3^	45	43	32
9	4.0 × 10^2^	2.4 × 10^2^	47	23	nd	2.0 × 10^2^
10	4.4 × 10^2^	1.9 × 10^3^	45	25	nd	76

Mean*	2.3^ab^	2.37^ab^	2.47^a^	1.85^b^	0.87^c^	1.83^b^

*log_10_; means with the same letter are not significantly different (*P* ≤ 0.05).

**Table 4:** Counts of coliforms (cfu/cm^2^) contaminating *rutab* samples.

Sample	*Khulas*	*Um Ruhaim*	*Shahal*	*Hilali*	*Tiar*	*Megnaz*
1	83	39	37	n.d.	91	99
2	46	9.1 × 10^2^	43	1.7 × 10^2^	nd	61
3	n.d.	n.d.	n.d.	7	nd	30
4	4	71	41	9	42	nd
5	n.d.	2	n.d.	2	35	86
6	n.d.	10	20	20	28	nd
7	3.0 × 10^2^	n.d.	9	n.d.	nd	32
8	n.d.	1.1 × 10^2^	41	n.d.	39	93
9	n.d.	n.d.	n.d.	11	nd	11
10	n.d.	2	2	2	37	10

n.d. = not detected.
